# A Simple, Fast and Portable Method for Electrochemical Detection of Adenine Released by Ricin Enzymatic Activity

**DOI:** 10.3390/toxins13040238

**Published:** 2021-03-26

**Authors:** George Oliveira, José Maurício Schneedorf

**Affiliations:** Department of Biochemistry, Federal University of Alfenas, Alfenas MG 37130-000, Brazil; zema@unifal-mg.edu.br

**Keywords:** ricin, square wave voltammetry, kinetic analysis, herring sperm DNA, depurination reaction

## Abstract

International authorities classify ricin toxin present in castor seed as a potential agent for use in bioterrorism. Therefore, the detection, identification, and characterization of ricin in various sample matrices are considered necessary actions for risk assessment during a suspected exposure. This study reports a portable electrochemical assay for detecting active ricin based on the adenine electro-oxidation released from herring sperm DNA substrate by its catalytic action. Also, kinetic parameters were calculated, and the values were *K_m_* of 3.14 µM and *K_cat_* 2107 min^−1^. A linear response was found in optimized experimental conditions for ricin concentrations ranging from 8 to 120 ng/mL, and with a detection limit of 5.14 ng/mL. This proposed detection strategy emphasizes the possibility of field detection of active ricin in food matrices and can be applied to other endonucleolytic activities.

## 1. Introduction

Ribosome-inactivating proteins (RIPs) are protein toxins extracted from bacteria or plants that have a similar mechanism of action, inactivating ribosomes by catalytically removing a specific adenine-4324 from the 28S rRNA subunit [1]. The RIPs can be classified into type I, a single-chain catalytic polypeptide, or type II, a catalytic and cell-binding chain [2,3]. The single-chain type III RIP has also been proposed, is synthesized as a proenzyme, and requires the removal of an internal peptide bond to become active [4,5]. Members of the type II RIP family are divided into non-toxic (e.g., ebulin, nigrin and pulchellin) and toxic (e.g., ricin, abrin, volkensin, stenodactylin, kirkiin and other plant toxins and the bacterial Shiga and Shiga-like toxins) [6,7,8,9].

Ricin is a toxic protein found in the endosperm of castor seeds (*Ricinus communis* L.). Ricin has gained recent attention from governments and the international scientific community due to the possibility of its use in poisoning, with a lethal LD_50_ dose of only 5 µg/kg in mice (inhalation), manifestations up to 4 h after inhalation, and irreversibility of the lethal condition between 6 to 12 h [10,11]. Ricin is considered one of the most potent toxins of plant origin, with an inactivating action on ribosomes. Studies have been reported an estimated lethal dose around 5–10 µg/kg in humans, both inhaled or injected, and about three orders of magnitude less toxic when taken orally [10]. Ricin is classified alongside the botulinum toxin as a biological agent of risk class III by the Health Biosafety Commission/MS, and Category B as a bioterrorism agent by the Atlanta CDC (Center for Disease Control and Prevention, GA, USA) [12,13], and is a controlled chemical under Schedule 1A of the Chemical Weapons Convention (CWC) [14]. In this context, the toxin arouses the interest of terrorist organizations, given the low cost of growing the plant, easy extraction of the protein, and good stability of the protein [15]. Thus, early detection of the active toxin is essential for developing appropriate countermeasures [16], justifying researchers’ efforts to obtain faster and more sensitive ricin detection tests [17].

Castor bean also has a broad spectrum of industrial uses and, in this sense, a fast identification of ricin traces without an expensive setup should be pursued. Castor cake, which is the remaining industrial by-product generated after the extraction of oil from castor beans, is capable of causing intoxication and death due to the presence of ricin [18]. Castor by-products are present in the industry of plastic polymers and polyamides, mono and diesters of fatty acids, biofertilizer, fungicide, pesticide, cosmetic, and pharmacological industries (purgative, antineoplastic, anti-inflammatory, and anti-rheumatic). Furthermore, a global market valued at US $1470 million [19] is expected for the biodiesel industry that uses castor by-products and castor oils (ricinoleic acid) by 2025, strengthening the importance of detecting and characterizing ricin activity traces.

Several approaches have been reported in the literature for detecting ricin and, according to the classification proposed by Bozza et al. [16], the methods can be differentiated from those that detect biologically active ricin and those that do not. Hence, it is of fundamental importance that a ricin detection method can distinguish between inactive and active forms of ricin, and its potency for adequate emergency actions, forensic analysis, and therapy [20].

Among the main analysis strategies unable to detect ricin activity, we highlight those that combine sample enrichment steps (using specific antibodies to ricin, aptamers, or sugar-conjugated materials) with improved detection technologies, such as surface plasmon resonance [21,22,23,24,25], polymerase chain reaction (PCR) [26] or mass spectrometry (MS) [27,28]. These methods also present some drawbacks regarding the need for laboratory infrastructure, a high cost for the equipment setup, several compounds involved, and the difficulty to allocate the equipment close to the site of the toxin exposure [4]. They may also involve some additional effort prior the analysis, such as sample enrichment, modification or even derivatization of the analyte [16].

Bioassays can measure the toxicity of ricin using laboratory animals [16] or by induced cytotoxicity in cultured cells [29,30]. However, they demand a long incubation time, in addition to the need for specific and costly infrastructure.

Although ricin’s *N*-glycosylase activity is commonly assessed with ribosomal inactivation protocols, the phytotoxin can also be described as a polynucleotide: adenosine *N*-glycosylase [31,32], due to the clearance of RNA and DNA substrates containing alternative adenosines [16]. Thus, monitoring the adenine released from the catalytic action of ricin can provide a convenient means of evaluating the catalytic activity of ricin [2,3,33,34,35]. In our experiments, we have used herring sperm DNA (hsDNA) as a substrate due to its simplicity of acquisition and use, besides its chemical stability and a significant low cost, as compared to synthetic oligonucleotides. As first reported by Barbieri et al., 1997, and later by Heisler et al., 2002, and Bevilacqua et al., 2010, hsDNA can be considered a good substrate for ricin [3,32,34].

Some methods detecting ricin activity are limited by technical disadvantages such as the analyte modification for spectroscopic detection, the derivatization of samples for chromatographic measurements, and the specific infrastructure and high cost. Besides, they present measurement difficulties in translucent media, such as in the presence of ribosome suspensions or complex matrices (liquids, biological material, other suspensions, or powdery extracts), without pre-purification of the sample before the detection of the analyte.

In this sense, electrochemical techniques have been proposed as a promising alternative due to some advantages over other methods, such as low cost, quick response, the relative simplicity of construction, small dimensions of the devices, small volume of the test sample, and high sensitivity [36]. Electrochemical techniques have also demonstrated outstanding potential for detecting and quantifying nitrogenous bases, especially those that quantify adenine [37,38,39,40,41].

**Figure 1:**
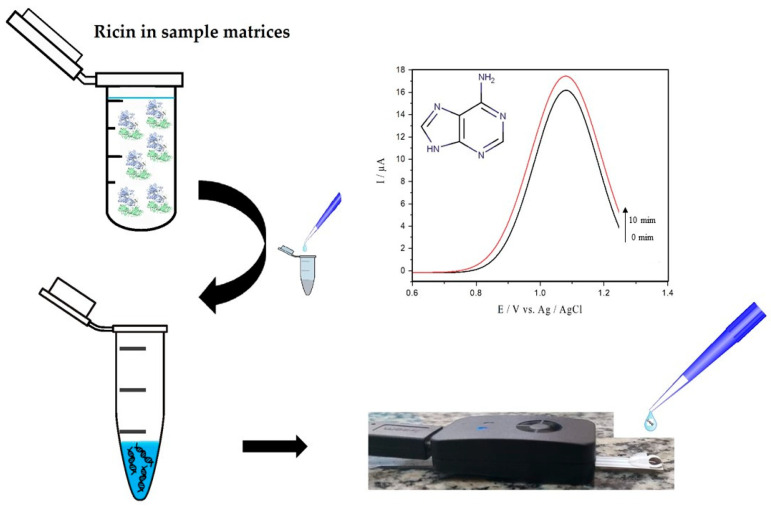
Protocol for detecting active ricin using the herring sperm DNA (hsDNA) substrate.

Here we report the electrochemical method of square wave voltammetry (SWV) to the detection of adenine released by ricin enzymatic activity. The method is focused on real-time traces of adenine released from hsDNA by oxidation of the analyte on the surface of an unmodified commercial printed carbon electrode (screen printed electrodes, SPE), as illustrated in Figure 1. The approach was centered on optimizing SWV parameters and experimental conditions for trace detection of active ricin in solution. The results were obtained both in pure and complex samples, the latter containing egg white or skimmed milk. As far as we know, this is the first report of the detection of adenine released after hsDNA depurination catalyzed by ricin by electroanalysis.

## 2. Results

### 2.1. Square Wave Voltammetry Method for Adenine Detection

The detection and quantification of nitrogenous bases, especially the techniques that quantify adenine, is frequently reported in the literature [36,38,40]. SWV was chosen in this work due to its high selectivity and sensitivity for adenine determination [42]. Square wave parameters were evaluated to obtain the highest signal-to-noise ratio for adenine oxidation [43,44]. The dependence of the peak current on square wave parameters was studied in the range of 10–100 mV of amplitude, 1–10 mV of step potential, and 5–100 Hz, by fixing two of these parameters at a constant value while measuring the other. The optimal values were 50 mV, 5 mV, and 100 Hz for amplitude, step potential, and frequency.

**Figure 2:**
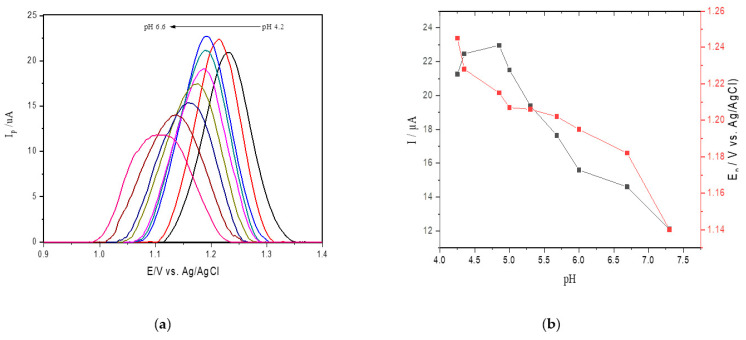
(**a**) Square wave voltammetry (SWV) for oxidation of 25 μM of adenine at different pH values (4.2–6.6) at the screen printed electrodes (SPEs) in acetate buffer; (**b**) anodic peak current obtained changing pH values.

The influence of pH on the SWV current for adenine oxidation at SPE was studied in acetate sodium buffer solution in the pH range from 4.2 to 6.6 (Figure 2a). Figure 2b shows an oxidation peak current for adenine with increasing pH values from 4.2 to 4.6, followed by a gradual decrease in faradaic currents for pH values ranging from 4.6 to 6.6. Therefore, considering that the highest peak current was obtained at pH 4.6 in sodium acetate buffer and that the value approaches the ideal pH of catalysis, this value was selected for the reactions catalyzed by ricin in hsDNA [45].

**Figure 3:**
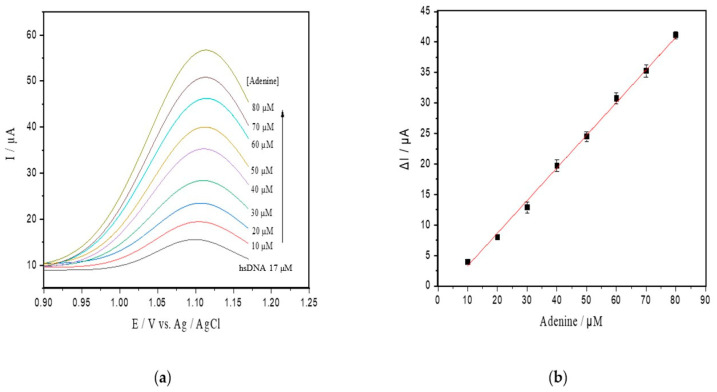

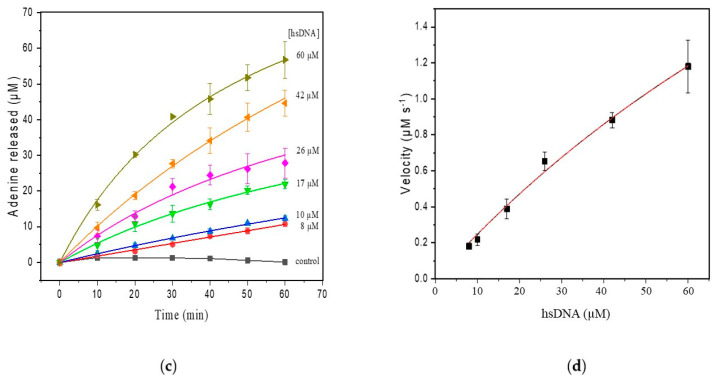
(**a**) SWV with varying concentrations of adenine up to 80 µM (pH 4.6) in the hsDNA sample. The arrow represents increasing adenine concentrations; (**b**) analytical curve of adenine; (**c**) progressive curve of adenine released after depurination reaction from hsDNA (8–60 µM) catalyzed by ricin at 100 ng/mL; (**d**) kinetic curve fitted for ricin catalysis on the hsDNA substrate.

Under the optimal conditions attained, an analytical curve was obtained by successive additions of a standard adenine solution to the supporting electrolyte solution containing hsDNA at 17 µM (Figure 3a). The peak currents for adenine doped in hsDNA solution increased linearly with a concentration range of up to 80 µM, and a detection limit of 0.18 μM (S/N = 3, Figure 3b) was found. This relationship resulted in the following linear equation: Ip (μA) = 0.492 ± 0.014 C (μM) + 0.003 ± 0.0002 (R^2^ = 0.996).

### 2.2. Detection of Active Ricin by Depurination of hsDNA

Apparent steady-state kinetics was investigated for ricin *N*-glycosylase activity at varying hsDNA substrate concentrations (8–60 µM) (Figure 3c). In our experiments, we used the hsDNA as this was previously reported as an alternative in ricin depurination tests [3,32,34]. The kinetic parameters calculated by fitting initial rates to the Michaelis–Menten model was characterized by a *K_m_* of 3.14 µM and a *V_max_* of 4.8 µM/min (Figure 3d). The turnover frequency (*k_cat_*) was found to be 2107 min^−1^.

**Figure 4:**
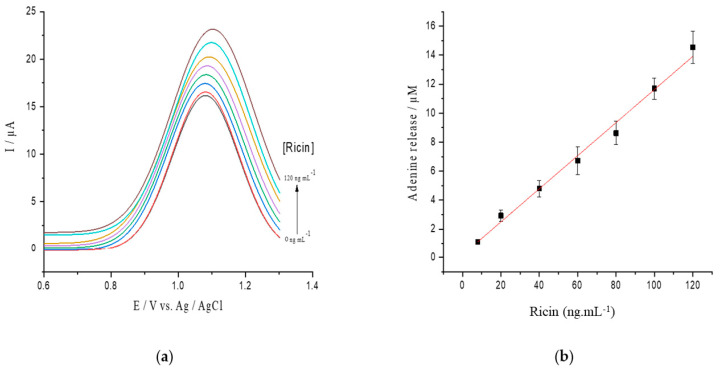
(**a**) SWV voltamograms of hsDNA treated with increasing concentrations of active ricin from 0 to 120 ng/mL; (**b**) released adenine after depurination from hsDNA catalyzed by different ricin levels.

To verify that the depurination reaction and adenine release are related to the ricin concentration, a calibration curve was tested between 0 and 120 ng/mL ricin on the hsDNA substrate at a concentration of 17 µM (Figure 4a). A linear correlation was observed in a range from 0 to 120 ng/mL of ricin concentrations, and the calibration curve was established as ΔIp (μA) = 0.114 ± 0.004 C_ricin_ (ng/mL) + 0.2163 ± 0.067 (R^2^ = 0.995) (Figure 4b) with the detection limit (LOD) estimated to be 5.14 ng/mL.

### 2.3. Analysis of Ricin in Spiked Samples

To evaluate the practical applicability of the proposed detection, active ricin was detected in different samples of spiked food, and with concentrations of 10 and 60 ng/mL of ricin. Considering that ricin may be ingested from contaminated beverages and foods, drinking water, hen egg and skimmed milk samples were chosen as sample matrices. Recovery values of spiked samples were determined from a calibration curve of ricin obtained in the assay buffer. Also, samples of drinking water, hen egg, and skimmed milk without ricin were tested as controls. The results of the recovery experiments are shown in Table 1.

As shown in Table 1, the method can be applied effectively to several matrix samples, and recoveries were calculated at 109.0% and 102.8% for drinking water, 81.0% and 95.2% for milk, and 58.0% and 78.2% for egg in concentrations of 10.0 and 60.0 ng/mL of ricin, respectively. Standard deviation values below 5% were found in the negative control, showing low interference from the food matrix in the hsDNA depurination.

## 3. Discussion

In this study, we developed and validated a straightforward method with the potential to be used in the field together with portable devices aiming to detect the glycosylase activity of ricin and other RIPs. The method is based on the electro-oxidation of hsDNA as the only added compound, and it was developed based on three basic criteria: speed for delineating the catalytic activity, no further necessity for modification or pre-concentration of the analyte, and the applicability for detection in complex matrices.

As shown in Table 1, the determination of active ricin based on hsDNA depurination in all three enriched and complex samples (drinking water, hen egg, and skimmed milk) showed acceptable results compared to those reported elsewhere [46]. The recovery values for active ricin from complex samples were greater than 81% and 58% for milk and egg samples, respectively. These results suggest that skimmed milk and hen egg samples may contain components that interact with ricin, partially inhibiting its activity against the hsDNA depurination [47]. However, the detected value for the active ricin was higher than the detection limit found in the assays containing buffer only.

As compared to well-known techniques usually found for ricin activity, the electrochemical technique described has some advantages, including no requirement for radioactive compounds [48], analyte modification for colorimetric detection [3], or derivatization of samples, as needed in chromatographic processes [2]. Furthermore, the voltammetric procedure does not require pre-purification before detection. It can realize direct measurements, unlike luminescence techniques [33,49], and can be used in non-translucent media, such as in the presence of ribosome suspensions, or a complex matrix. Besides, the possibility of field measurements of ricin activity stands out as amperometric systems are known to be easily miniaturized and customized [50].

Among the methods for measuring ricin activity, oligonucleotide depurination, fluorescence, molecular absorption, electrochemiluminescence, high-pressure liquid chromatography (HPLC), and mass spectrometry (MS) can be referenced [2,3,34,51]. The approaches involving HPLC and MS, or both, are the most used for this purpose due their high specificity and resolution [4,28,52]. As an advantage, they have a detection limit as low as 0.6 ng/mL [35,53,54]. As an example, Wang et al., 2016, and Feldberg et al., 2021, reported a HPLC–MS assay for ricin detection conjugated with polyclonal antibody [35] or lectin affinity capturing of ricin by lactose-agarose beads [28] recently developed to improve specificity and to offer more accurate quantification.

As an alternative for the HPLC–MS procedure, new methods have been proposed, improving the possibility for use on-site. The detection strategies involve measurement by fluorescence or colorimetric, and surface-enhanced Raman spectroscopy (SERS) [4,46,55,56]. In a previous report, a specific nanoprobe involving nanoparticles and quantum dots was designed to detect active ricin [55]. Its main advantage is the capture of ricin in complex matrices by monoclonal antibodies, with a LOD of 7.46 ng/mL. It also involves the immobilization of specific double-stranded oligodeoxynucleotide substrates to the nanoparticles, and fluorescence suppression as a quantification technique. Similar to that strategy, gold nanoparticles were conjugated with oligodeoxynucleotide consisting of homoadenin, with subsequent formation of the homoadenin/coralyne complex as a result of specific depurination of the poly (21dA) substrate, and with ricin detection by visual inspection or UV-VIS absorbance [46]. From the same research group, the substrate poly (21dA)-conjugated gold nanoparticles was used to constitute a specific chip for enhanced surface Raman spectroscopy [56]. Although the methods mentioned above involve the detection of ricin based on its *N*-glycosylase activity on oligonucleotides, all of them require several steps that with analytical care, e.g., the magnetic removal of nanoparticles added to the matrix to capture ricin before detection by biosensors, conjugation with nanoparticles, or colorimetric indicators.

On the other hand, there are some RIPs with potential risks for bioterrorism that also exhibit the same *N*-glycosylase activity as ricin [57], e.g., abrin and Shiga toxin [58]. Therefore, the electrochemical assay described here for detecting ricin is not able to distinguish between the different toxins that cause the release of adenine in nucleic substrates. Thus, to achieve this goal, further steps must be added to verify specificity, e.g., the use of an additional ricin capture approach. Even so, the procedure described could be useful for an immediate screening test for ricin and other RIP toxins in emergencies.

Although some methods for ricin detection can be more specific due to the additional step of its capture [4,28,35,46,55,56], the *N*-glycosylase activity of ricin by SWV comprises both fewer steps and chemical compounds, and uses relatively low-cost equipment. In this regard, the toxin can be detected in a broad range of instruments, from lab bench or battery-powered potentiostats to interfaced mobile devices joined to commercially portable instruments (WiFi, BlueTooth), or even microcontroller boards [59]. Besides, the possibility of field measurements for ricin activity stands out as amperometric systems are known to be easily miniaturized and customized [50].

Other advantages of the electrochemical approach comprise the use of inexpensive unmodified carbon electrodes of paper or solid-phase base, and a short-time interval for detection. In this respect, the SWV approach for ricin activity was able to be accomplished in up to 10 min (Figure 3c), differing from other methods that take additional steps for sample preparation and ricin capture (30 to 120 min), as reported above [28,35,55]. Hence, these overall benefits of the SWV method are attractive for the rapid detection of active ricin in suspected samples in practical situations, as in the castor oil industry, or in the countermeasures involved in bioterrorism.

## 4. Conclusions

In this study, we have used square wave voltammetry to detect ricin by monitoring the adenine released by ricin catalytic action in suspected samples. Compared to other techniques, the method is simple, uses unmodified carbon paste or SPE electrodes, did not require any sample preparation, efficiently detected ricin in a nanomolar range, and can be easily portable for field detection.

## 5. Materials and Methods

### 5.1. Reagents and Solutions

All reagents were of analytical grade, and the solutions were prepared with Milli-Q water. Adenine and herring sperm DNA (hsDNA) were obtained from Sigma-Aldrich (St. Louis, MA, USA). A 50 mM sodium acetate buffer solution was prepared, adjusting the pH values either with 0.1 M NaOH or 0.1 M HCl. Graphite powder (99.95%, 325 mesh) were obtained from Sigma-Aldrich. The denatured hsDNA was obtained following Bevilacqua et al. [34]. Briefly, native hsDNA was dissolved in acetate buffer (pH 4.6), heated in a water bath at 95 °C for 5 min, followed by cooling on ice for another 5 min. Intact ricin was obtained with a purity exceeding 95% by SDS-PAGE analysis after extraction from castor beans following standard procedures [60].

All trials involving ricin were conducted by adopting techniques and working practices following the risk class [61].

### 5.2. Instrumentation and Data Analysis

All experiments were conducted with a portable potentiostat Sensit Smart (PalmSens, GA, The Netherlands). The assays were conducted with a commercially printed carbon electrode (screen printed electrodes, SPE), which consists of an arrangement containing three electrodes deposited on the same ceramic alumina plate (AC1.W4.R1, by PalmSens).

For the electrochemical assay, square wave voltammetry (SWV) was employed in acetate buffer (50 mM, pH 4.6) with experimental conditions from 50 Hz of frequency, a potential window from +0.6 to +1.4 V, sweep increment (∆Es) of 5 mV, and amplitude of 50 mV.

Data were obtained at a minimum of triplicates and represented by mean ± standard deviation. The results of the adjustments obtained with a *p*-value less than 0.05 were accepted as significant after treatment of the data with the aid of the statistical package of free distribution R (version 3.21). The quality of linear mathematical adjustments was assessed by a simple comparison of Pearson’s correlation coefficient (R) values and dispersion parameters. All the experiments were conducted in triplicate.

### 5.3. Detection of Ricin Activity

Adenine released during the depurination activity of holoricin on hsDNA was measured by SWV. For the activity assay, varying concentrations of denatured hsDNA solubilized in 50 mM sodium acetate buffer, pH 4.6, were incubated with ricin (100 ng/mL) under mixing, at a total reaction volume of 3 mL and 25 °C. At regular intervals, a 100 µL aliquot was removed from the reaction system and added to the SPE for reading. The apparent kinetic parameters were calculated based on the classical Michaelis–Menten equation ν = *V_max_* × [S]/(*K_m_* + [S]), where ν is the initial velocity, *V_max_* is the maximal reaction velocity, [S] is the concentration of the substrate, and *K_m_* is the Michaelis–Menten constant.

### 5.4. Ricin Activity in Food Matrices

Some tests were carried out to verify the possibility of interference of compounds inherent to the complex matrices, such as hen egg and skimmed milk, to detect ricin. To minimize the effects of the food matrix on the assay’s performance, hen egg and skimmed milk samples were diluted to 1:3 in sodium acetate buffer (50 mM, pH 4.6). In this assay, ricin aliquots were added to liquid egg and skimmed milk samples and incubated for 10 min at 25 °C. Then, 400 µL of the mixture was added to a reaction system containing 17 µM of denatured hsDNA solubilized in the buffer, and under stirring for 30 min at 25 °C. The ricin concentration detected (recovery) in each sample was calculated by comparing the determined concentrations with the added one.

## Figures and Tables

**Table 1:** Determination of active ricin spiked in different matrices.

Sample	Spike (ng/mL)	Detected (ng/mL)	Recovery (%)
Drinking water	10.0	10.9	109.0
	60.0	61.7	102.8
Skimmed milk	10.0	8.1	81.0
	60.0	57.1	95.2
Hen egg	10.0	5.8	58.0
	60.0	46.9	78.2

## Data Availability

Not applicable.
